# Effects of Inhaled Citronella Oil and Related Compounds on Rat Body Weight and Brown Adipose Tissue Sympathetic Nerve

**DOI:** 10.3390/nu7031859

**Published:** 2015-03-11

**Authors:** Irmanida Batubara, Irma H. Suparto, Siti Sa’diah, Ryunosuke Matsuoka, Tohru Mitsunaga

**Affiliations:** 1 Biopharmaca Research Center, Bogor Agricultural University, Jl. Taman Kencana No 3, Bogor 16128, Indonesia; E-Mails: irma.suparto@yahoo.com (I.H.S); diah.ss@gmail.com (S.S.); 2 Department of Chemistry, Faculty of Mathematics and Natural Sciences, Bogor Agricultural University, Darmaga Campus, Bogor 16680, Indonesia; 3 Primate Research Center, Bogor Agricultural University, Jl. Lodaya 2 No 5, Bogor 16151, Indonesia; 4 Department of Anatomy, Physiology and Pharmacology, Faculty of Veterinary Medicine, Bogor Agricultural University, Darmaga Campus, Bogor 16680, Indonesia; 5 Faculty of Applied Biological Science, Gifu University, 1-1 Yanagido, Gifu 501-1193, Japan; E-Mails: mr.e0729sky.fantasy@gmail.com (R.M.); mitsunaga@gifu-u.ac.jp (T.M.)

**Keywords:** citronella oil, *R*-(+)-citronellal, β-citronellol, inhalation

## Abstract

Citronella oil is one of the most famous Indonesian essential oils, having a distinctive aroma. As with other essential oils, it is crucial to explore the effects of inhalation of this oil. Therefore, the aim of this research was to elucidate the effects of inhalation of citronella oil and its components isolated from *Cymbopogon nardus* L. (Poaceae), Indonesian local name: “*Sereh Wangi*” on the body weight, blood lipid profile, and liver function of rats, as well as on the sympathetic nerve activity and temperature of brown adipose tissue. Sprague-Dawley male adult rats fed with high fat diet (HFD) were made to inhale citronella oil, *R*-(+)-citronellal, and β-citronellol for five weeks, and the observations were compared to those of HFD rats that were not subjected to inhalation treatment. The results showed that inhalation of β-citronellol decreased feed consumption. As a consequence, the percentage of weight gain decreased compared with that in control group and the blood cholesterol level in the β-citronellol group was significantly lowered. Concentration of liver function enzymes were not significantly different among the groups. In conclusion, inhalation of citronella oil, specifically β-citronellol, decreased body weight by decreasing appetite, without any marked changes in liver enzyme concentrations.

## 1. Introduction

Indonesia is a country with many tropical plants, including medicinal plants. These plants contain certain essential oils having distinctive scents or aromas. One of the most famous essential oils in Indonesia is *Minyak Sereh Wangi* or citronella oil. This oil is produced from *Sereh Wangi* (*Cymbopogon nardus* L, Poaceae) plants, found in Bintan Island, Indonesia [1].

*Sereh Wangi* is traditionally applied externally as a component of health and personal products, such as soaps, anti-mosquito oils, candles, and pain-reliever oils. Through external application, the components of the essential oil enter the body through the skin or the respiratory system.

Reports on the mechanism underlying the inhalation of essential oils and their compounds as aromatherapy remain limited. It was reported that olfactory stimulation by grapefruit oil in rats could affect autonomic nerves, increase lipolysis, heat production, and reduce appetite and body weight [2]. In contrast to the mechanism of grapefruit oil, lavender oil affects the autonomic nerves, decreases lipolysis and heat production, but increases appetite and body weight in rats [3]. Information on the anti-obesity effects of inhalation of citronella oil in rats will be beneficial.

**Figure 1:**
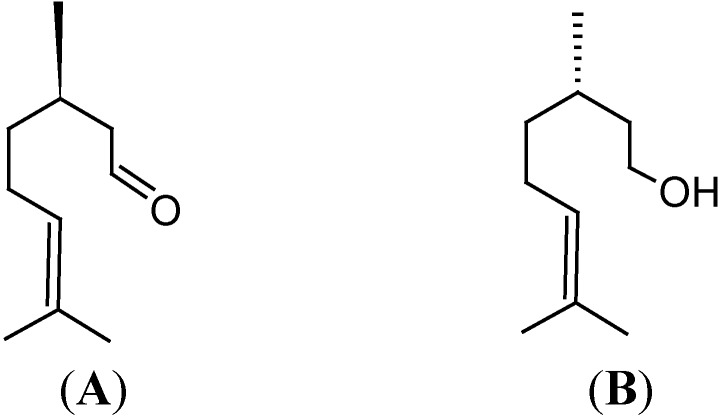
Structures of *R* citronellal (**A**) and β-citronellol (**B**).

Obesity can be a health concern because it can interfere with bodily functions and lead to the development of several serious diseases, such as hypercholesterolemia, high blood pressure, coronary heart disease, and diabetes. It also increases the risk of cancer [4]. Thus, it is necessary to treat or prevent obesity, to lower the risk of these diseases. The effects of citronellal and citronellol (Figure 1), as the main components of citronella oil [5], were studied by evaluating body weight, feed consumption, fecal excretion, parasympathetic nerve activity, and brown adipose tissue temperature response in rats.

## 2. Method and Experimental Details

### 2.1. Isolation of Citronella Oil from C. nardus

*C. nardus* leaves used in this experiment were collected in the morning from the Conservation and Cultivation Unit of Biopharmaca Research Center, Bogor Agricultural University, Bogor, Indonesia in July 2013. The sample was identified by Herbarium Bogoriense, Cibinong, Indonesia and deposited in Biopharmaca Research Center Bogor Agricultural University (No. PSB-IPB 0042013). The leaves (500 g fresh leaves with 5 L water) were distilled by water distillation. Distillation period was 1 h, and the essential oil yield 2% citronella oil (v/w) after dried by anhydrous sodium sulfate. The citronella oil obtained was analyzed by GC-MS.

### 2.2. GC-MS Conditions

Citronella oil and its fractions were separated using column DB-5MS (Agilent, California, CA, USA, 0.25 mm × 30 m) with helium gas (flow rate 42 mL/min). The injector temperature was 80 °C and detector temperature was 250 °C. Component separation was performed with a temperature program, starting at 80 °C for 5 min, with an increase in temperature at a rate of 5 °C/min until it reached 250 °C, following which the temperature was kept constant for 45 min. The Electron Impact-Mass Spectrometry (EI-MS) analysis was performed at 70 eV, and a split ratio of 50:1. Mass Spectrometry data were collected in the range of 40–500 *m/z*. The MS spectrum was then compared with the NIST data, manual interpretation of mass spectra based on comparisons with the literature, and Kovats retention index. The Kovats retention indices were calculated using n-alkanes C8–C20 and the experimental values were compared with those reported in literature.

Kovat retention (KIs) was determined as follows: citronella oil was injected to Agilent GC Seri 7890 gas chromatograph equipped with Agilent MS seri 6950. A fused silica capillary column (30 mm × 0.25 mm i.d.) with an HP-5ms (J & W Scientific, California, CA, USA, 5% phenyl 95% polydimethylsiloxane) bonded phase was used to separate the oil constituents. Direct injection of 2.0 μL of citronella oil with helium (flow rate 1.0 mL/min) as a carrier gas (100:1 split-vent ratio), oven temperature held isothermal at 40 °C for 2 min and then programmed to increase at 10 °C/min to 280 °C gave complete elution of all peaks. The injector temperature was 250 °C. Essential oil constituents were identified based on retention time and by co-injection with authentic compounds (*n*-alkanes C8–C20), and the relative peak area to total peak areas were used to determine the composition.

### 2.3. In Vivo Analysis

The male adult Sprague-Dawley rats were housed in groups of three rats per cage. Feed and water were given *ad libitum*. Rats were then divided into four groups, with six rats in each group. The first group consumed a high fat diet and was not subjected to inhalation treatment. Treatment groups 2, 3, and 4 received a high fat diet and were treated with the inhalation of citronella oil, R citronellal (TCI, Tokyo, Japan) and β-citronellol (TCI, Tokyo, Japan) diluted 100× in water, for 35 days. During the treatment period, all rats were monitored every week in terms of their body weight, daily weight of feed consumption, and fecal-urine excretion (based on bedding weight) every 3 days. After 5 weeks of treatment, animals were fasted overnight and sedated by using a combination of ketamine and xylazine, with a dosage of 80 mg/kg and 10 mg/kg body weight, respectively. Blood was collected intracardially to determine the lipid profile (cholesterol and triglyceride) and the concentrations of liver enzymes alanine transaminase (ALT) and aspartate aminotransferase (AST). Samples were assayed enzymatically using specific kits manufactured from Biolabo SA (Maizy, France). Animals were then euthanized with sodium phenobarbital (20 mg/kg body weight), and adipose tissue from the scrotal and mesenteric areas were weighed. All procedures with animals were approved by the animal ethics committee of Bogor Agricultural University No. 04/2013 on 29 April 2013.

### 2.4. Electrophysiological Recording

#### 2.4.1. Animals

Male Wistar rats, weighing 270–310 g, were used. Rats were housed in a room maintained at 25 °C ± 1 °C and illuminated for 12 h (07:00 am to 19:00 pm) every day. Feed and water were freely available. Rats were adapted to the environment for at least 1 week prior to the experiment.

#### 2.4.2. Measurement of Sympathetic Nerve Activity in Brown Adipose Tissue

Brown Adipose Tissue Sympathetic Nerve Activity (BSNA) was determined in the urethane-anesthetized rats. Each rat was anesthetized with urethane (1 g urethane/kg body weight) prior to surgery. Each rat was placed in the prone position and a small incision made above the scapula to separate the brown adipose tissue from the muscle. Five efferent nerves were identified and one of the five nerve branches was dissected. An isolated nerve was placed between a pair of silver wire electrodes. The recording electrodes were immersed in a mixture of warm Vaseline and liquid paraffin oil to prevent drying of the nerves and for electrical insulation. Each rat was allowed to stabilize for 30–60 min after being placed on the recording electrodes.

The original signal of the efferent discharges from the electrodes was amplified and filtered using a Bioelectric Amplifier ER-1. The amplified signal was converted to digital signals by Power Lab (AD Instruments, Colorado, CO, USA). Then, these signals were recorded on a computer as spike histograms through recording software. Spikes above a threshold voltage level set just above the background were counted by spike histograms. Throughout all the experiments, the quality of nerve recordings was continuously monitored by an oscilloscope. The amplification, filtration, and sampling rate settings of the signals from sympathetic nerves were held constant across all studies.

### 2.5. Experimental Protocol

Baseline measurements of BSNA were recorded 40 min prior to olfactory stimulation with the samples and water. Samples used were diluted 100× with water. A folded Kimwipe containing the sample was placed at the bottom of a paper cup, and the rat’s nose was placed inside the paper cup for 10 min. After the start of the olfactory stimulation, BSNA was recorded for 60 min.

### 2.6. Recording of Brown Adipose Tissue (BAT) Temperature

The temperature sensor was implanted into the subcutaneous space between the interscapular BAT. The temperature of BAT was recorded before, during, and after inhalation of citronella oil and its fractions.

### 2.7. Statistical Analysis

The experiment was laid out in a completely randomized design (CRD), and the data obtained were analyzed with analysis of variance (ANOVA) using SAS program at the confidence level of 95% (*α* level of 0.05). *P* values < 0.05 indicate a statistically significant difference. Another test used was the Duncan test.

## 3. Results and Discussion

**Figure 2:**
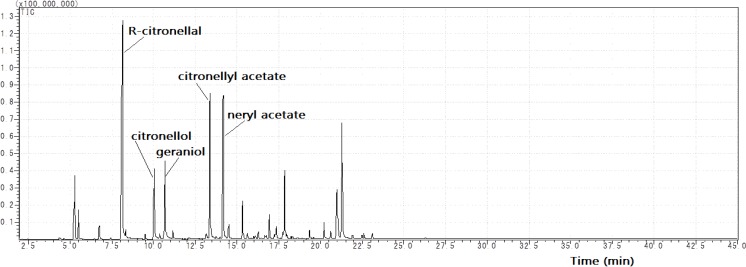
Total ion chromatogram of citronella oil.

The isolated citronella oil consisted of *R* citronellal (KIs 1157) as the major component (26%), along with neryl acetate (KIs 1365, 13%), citronellyl acetate (KIs 1273, 10.5%), geraniol (KIs 1250, 7.6%) and β-citronellol (KIs 1226, 6%). The total ion chromatogram of citronella oil is shown in Figure 2. The components of the citronella oil used in this study are the same as those seen in citronella oil from Togo, Bangladesh; the concentration of each component was the only difference [5]. *R*-(+)-citronellal and geraniol are two common compounds used for perfumery, therefore, citronella oils are an essential component of perfume [6]. In this study, citronella oils, R citronellal and β-citronellol, were used to identify the effects of the inhalation of the citronella oils and their components on rats.

There are several mechanisms by which a compound can act as a slimming drug. It can stimulate fat burning, block absorption of fat within a certain limit, and reduce appetite [7]. Inhalation treatment of citronella oil is suggested to inhibit appetite, eventually decreasing the body weight. Inhalation treatment or aromatherapy is believed to be an ancient practice that is increasingly preferred by consumers nowadays. This is due to its soothing and relaxing effects, which also have several benefits, such as alleviation of pulmonary problems, postpartum discomfort, and possible appetite reduction [6].

**Figure 3:**
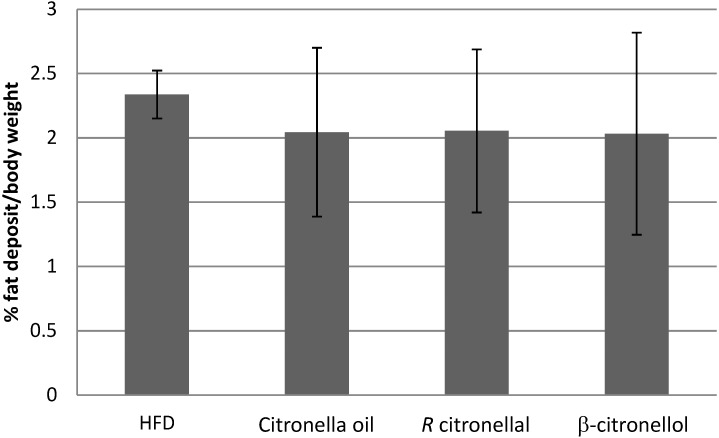
Percentage of adipose tissue to body weight of all animals in each group.

The results of the animal experiment are shown in Table 1. Rats treated with 1% β-citronellol for 5 weeks had the lowest percentage of weight gain compared with that observed in the other three groups. The average feed consumption and fecal-urine excretion weight (based on weight of bedding) were not significantly different in all groups (*p* > 0.05) (Table 1). There was no significant difference on the adipose tissue or fat collected from the scrotal and mesenteric areas between all groups. Animals receiving a high fat diet without inhalation treatment showed a trend of higher adipose tissue compared to the others (*p* > 0.05) (Figure 3).

**Table 1:** Average feed consumption, fecal and urine excretion, and percentage of body weight increase in all groups.

Treatment Groups	Feed Consumption (g/Animal)		Fecal & Urine Excretion (g/Animal)		% Increase in Body Weight Compared to Baseline
	Before Treatment	After Treatment	Before Treatment	After Treatment	
HFD (High Fat Diet)	20.00 ± 0.00 ^a^	19.05 ± 0.95 ^b^	18.92 ± 0.52 ^a^	14.40 ± 4.51 ^a^	34.41 ± 14.75 ^a^
HFD + citronella oil	20.00 ± 0.00 ^a^	18.34 ± 1.66 ^b^	18.65 ± 1.99 ^a^	15.75 ± 2.90 ^a^	30.12 ± 9.83 ^a^
HFD + R-citronellal	20.00 ± 0.00 ^a^	18.26 ± 1.74 ^b^	18.07 ± 1.91 ^a^	15.60 ± 2.47 ^a^	34.71 ± 15.21 ^a^
HFD + citronellol	20.00 ± 0.00 ^a^	15.49 ± 4.51 ^a^	17.75 ± 1.99 ^a^	12.28 ± 5.47 ^a^	25.96 ± 11.27 ^a^

HFD: high fat diet; superscript lower case letters indicate significance at *p* > 95%.

Concentration of blood cholesterol (Table 2) was significantly different between treatments and highest in animals receiving the R citronellal. The cholesterol concentration of the citronella oil group was not different in the HFD group even though it showed a more reduced cholesterol concentration (*p* > 0.05). β-citronellol gave the lowest blood cholesterol concentration.

**Table 2:** Blood cholesterol, triglyceride, alanine transaminase (ALT) and aspartate aminotransferase (AST) concentrations of all groups.

Groups	Cholesterol (mg/dL)	Triglyceride (mg/dL)	ALT (U/L)	AST (U/L)
HFD (High Fat Diet)	110.98 ± 23.75 ^ab^	56.71 ± 9.19 ^a^	52.04 ± 13.29 ^a^	45.31 ± 13.56 ^a^
HFD + Citronella oil	101.45 ± 27.90 ^ab^	59.71 ± 5.51 ^a^	40.60 ± 20.78 ^a^	54.70 ± 31.14 ^a^
HFD + R-citronellal	130.17 ± 13.40 ^b^	55.27 ± 10.12 ^a^	42.06 ± 11.19 ^a^	38.49 ± 13.31 ^a^
HFD + citronellol	89.95 ± 16.46 ^a^	70.53 ± 9.95 ^b^	47.43 ± 13.57 ^a^	46.64 ± 26.29 ^a^

HFD: high fat diet; superscript lower case letters indicate significance at *p* > 95%.

Total plasma cholesterol in animals inhaling β-citronellol showed a trend of the lowest concentration and with the highest triglyceride concentration (Table 2) (*p* < 0.05). Even though the concentration of all triglyceride were still in the normal value [8].

For liver function, treatment groups were not significantly different compared with control groups. This showed that the samples did not affect liver function based on the two parameters, ALT and AST (Table 2).

Inhalation of citronella oil, R citronellal and β-citronellol illustrated a trend to reduce body weight that is relevant with the decrease in the intake of feed and likewise lowered plasma cholesterol and triglyceride. These effects are related to the sympathetic nerve activity in brown adipose tissue (BAT) [2]. Sympathetic nerve activity suggests thermogenesis in BAT that converts fatty acids to fuel [9].

**Figure 4:**
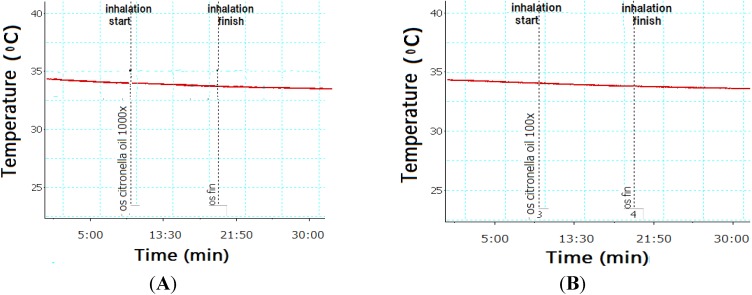
Brown adipose tissue (BAT) temperature of rat inhaling citronella oil at 1000× (**A**) and 100× (**B**) dilutions.

**Figure 5:**
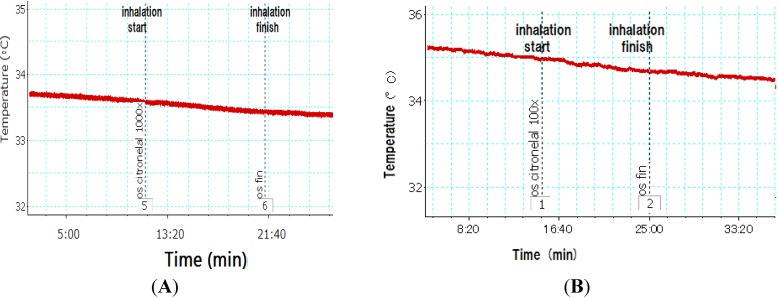
Brown adipose tissue (BAT) temperature of rat inhaling *R* citronellal at 1000× (**A**) and 100× (**B**) dilutions.

**Figure 6:**
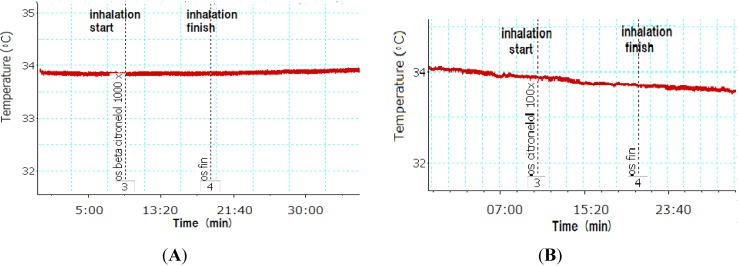
Brown adipose tissue (BAT) temperature of rat inhaling β-citronellol at 1000× (**A**) and 100× (**B**) dilutions.

The effect of inhaling citronella oil on BAT temperature in rats is described in Figure 4. The BAT temperature was not changed during and after inhalation compared to that before inhalation. When inhaling 1000× and 100× dilutions of citronella oils, the BAT temperature rate did not change compared to that before and after inhalation (Figure 4). Inhaling 100× *R*(+)-citronellal did not change the temperature of BAT. Five minutes after inhalation of 1000× *R*(+)-citronellal, a slight increase in the temperature was observed (Figure 5). BAT temperature also slightly increased when inhaling β-citronellol 1000× dilutions (Figure 6A), but with 100× no effect was observed (Figure 6B).

**Figure 7:**
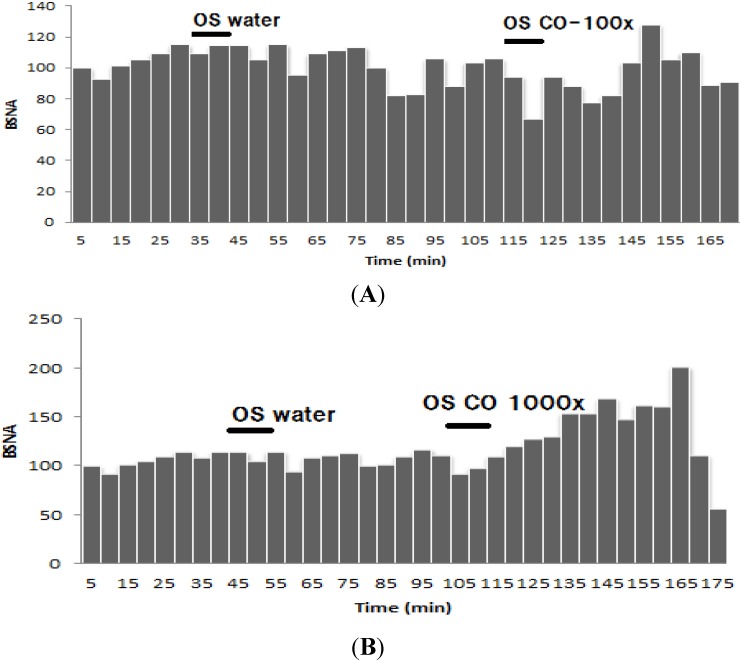
Effect of inhaling citronella oil at 100× (**A**) and 1000× (**B**) dilutions on brown adipose tissue sympathetic nerve activity (BSNA) (%).

The effects of inhalation of the diluted citronella oils on BAT sympathetic nerve activity (BSNA) are shown in Figure 7. The results showed that inhalation of 100 times diluted citronella oils had no effect on the activity of BAT sympathetic nerve, just like when inhaling water. However, inhalation of 1000× diluted oil could decrease the activity of BSNA but not at a level that is significantly different from that observed with the control.

**Figure 8:**
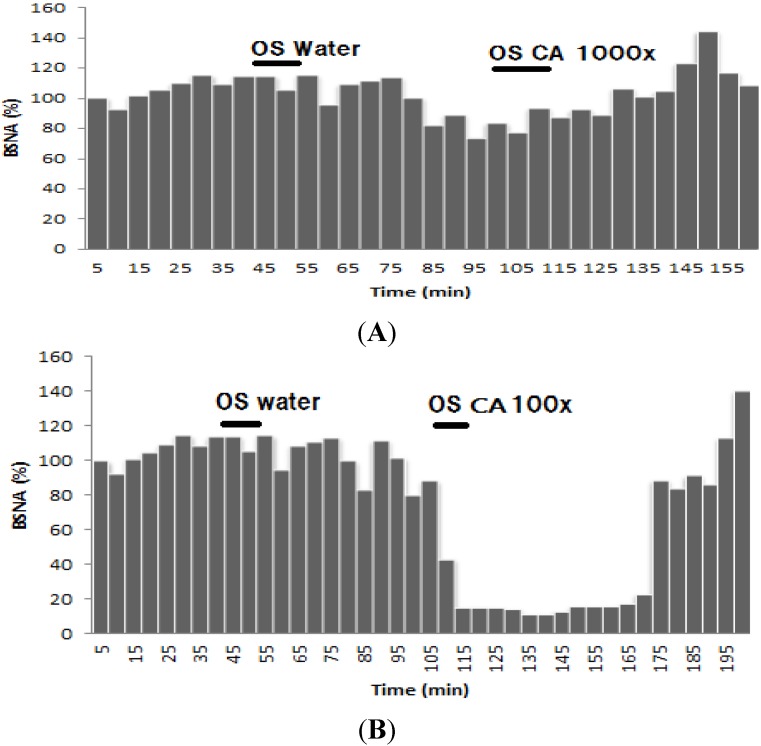
Effect of inhaling (R)-citronellal at 100× (**A**) and 1000× (**B**) dilutions on brown adipose tissue sympathetic nerve activity (BSNA, %).

**Figure 9:**
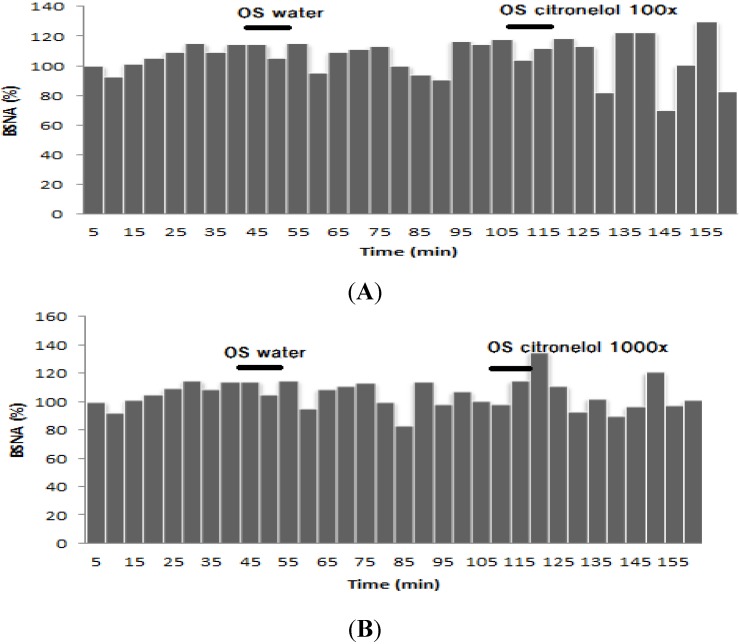
Effect of inhaling citronellol at 100× (**A**) and 1000× (**B**) dilutions on brown adipose tissue sympathetic nerve activity (BSNA, %).

Inhalation of R citronellal in 100× dilutions decreased 10% of the BSNA activity compared to the control. This effect lasted 60 min after inhalation ended, but not for 1000× diluted (*R*)-citronellal (Figure 8). Inhaling β-citronellol in both dilutions (100 and 1000 times) increased sympathetic nerve activity (Figure 9) but not significantly differently from that observed with water.

Citronella oil inhalation did not affect weight gain or feed consumption and did not change the lipid profile (Table 1 and Table 2, and Figure 3). Only a small decrease in weight gain was found in the citronella oil group. The triglyceride concentration in citronella oil group is higher than the HFD group but not significantly different. In addition, the cholesterol concentration in the citronella oil group is smaller compared to the HFD group (not significantly different, Table 2). The adipose tissue weight also decreased compared to that in the HFD group (Figure 3). Thermogenesis effect on the inhalation of the citronella oil could not be found (Figure 5) but the BAT sympathetic nerve activity increased after 30 min of inhaling the oils. These results are different from those seen with inhalation of *Zingiber zerumbet* essential oils, having zerumbone as the major component, which increases body weight by increasing feed consumption [10].

Among the different group, the lowest triglyceride level was found in R citronellal group. It could be easily understood that BAT sympathetic nerve activity could be decreased by the inhalation of R citronellal (Figure 8B). A decrease in the BAT sympathetic nerve activity is known to reduce triacylglycerol hydrolysis [8]. The lowest triglyceride level in the blood gave an increase cholesterol level in the blood (Table 2). BAT sympathetic nerve activity is also reported to be closely related to the feed intake [11]. When the sympathetic nerve activity decreases, the feed intake increases. This effect is still unclear in this research, since the feed consumption of R citronellal group was not significantly different with the HFD group.

Between the three oils, β-citronellol could decrease the body weight more effectively compared to the other groups. The lowest increase in the body weight could be due to the lowest feed consumption of the β-citronellol group (Table 1). This means that the β-citronellol could decrease the feed consumption as well as the body weight. The decreasing appetite of the rats inhaling β-citronellol occurred with the increase in sympathetic nerve activity (Figure 9). A decrease in appetite was observed when the sympathetic nerve activity was excited. On the other hand, the temperature of BAT after the inhalation of β-citronellol slightly increased (Figure 6). High fat diet on the animals induced hypercholesterolemia and increased body weight. In this study, cholesterol concentrations were lowered by inhaling β-citronellol in the animals that had the lowest increase in body weight. The triglyceride level in the blood of the β-citronellol group was the highest while the cholesterol level was the lowest.

## 4. Conclusions

Citronella oil from Bogor, Indonesia consists of *R* citronellal, nerol acetate, citronellol acetate, *cis* geraniol and β-citronellol as major components. No liver function was damaged by inhalation of citronella oil, *R* citronellal, and β-citronellol. Inhalation of citronella oil, specifically β-citronellal decreased weight gain by increasing the sympathetic nerve activity, which was related to decreased appetite. The cholesterol level also decreased in rats inhaled β-citronellol.
